# Elucidating the Environmental and Health Risks of Trace Element Pollution in Red Sea Fish from Nuweiba City, Aqaba Gulf, Egypt

**DOI:** 10.1007/s12011-024-04246-w

**Published:** 2024-06-28

**Authors:** Mohamed A. El-Shorbagy, Shimaa M. Abdel-Moniem, Mohamed H. Ghanem, Mohamed A. Embaby, Mohamed S. Kourany, Ahmed A. El-Kady, Mahmoud Mahrous M. Abbas

**Affiliations:** 1 Marine Biology Branch, Zoology Department, Faculty of Science, Al-Azhar University, Cairo, Egypt; 2 Water Pollution Research Department, Environmental and Climate Changes Research Institute, National Research Centre, Cairo, Egypt; 3 Food Toxicology and Contaminants Department, National Research Centre, Cairo, Egypt; 4 Food Science and Technology Department, Agriculture Faculty, Fayoum University, Fayoum, Egypt

**Keywords:** Arsenic, Children consumers, Mercury, Carcinogenic risks, Bioaccumulation factor, Contamination degree

## Abstract

Trace element bioaccumulation in marine organisms is a rising international issue due to possible health concerns for humans. Thirteen trace elements were analyzed in the sediment, water, and muscular tissue of Red Sea fish. Additionally, the average daily intake (EDI), the cancer risk (CR), the hazard index (HI), and the target hazard quotient (THQ) of those elements have been taken into consideration when evaluating any possible health concerns related to their consumption. All species presented quantifiable values in muscle for all the analyzed elements (arsenic (As), lead (Pb), copper (Cu), aluminum (Al), boron (B), iron (Fe), barium (Ba), manganese (Mn), nickel (Ni), cadmium (Cd), chromium (Cr), zinc (Zn), and mercury (Hg), except for Cd and Hg, being Fe and Zn the most accumulated elements in all species. Conversely, in water samples, most elements were undeleted except for aluminum, boron, iron, and zinc. All Red Sea fish, however, had concentrations of Zn, Ni, Fe, Cu, and Mn below the upper limit allowed, although most species had higher levels of As, Cr, and Pb (0.48 ± 0.83–5.10 ± 0.79, 1.97 ± 0.46–5.25 ± 0.67 and 2.12 ± 1.01–6.83 ± 0.93 µg/g, respectively).

The studied Red Sea fish showed contamination degrees (CD) of Mn, Cu, Fe, Ni, Zn, and Pb were ≤ 1, indicating minimal contamination, with As and Cr showing higher contamination degrees. However, the pollution index values (MPI-elements) can be represented according to ascending order: *Lethrinus ramak* < *Cephalopholis hemistiktos* < *Pagellus affinis* < *Trachurus japonicus* < *Cheilinus lunulatus* < *Siganus luridus* < *Parupeneus forsskali* < *Caesio suevica*. The study found that edible tissues are safe for human consumption, with HI values for children and adults less than ten, indicating negligible non-cancer hazards. However, fish consumption presents health risks due to chromium, lead, and arsenic, with THQ values several times greater than 1, and CR-Ni, CR-Cr, and CR-As values exceeding the acceptable 10^−4^ value in all studied species. This study provides critical insights into trace element contamination in marine fish species, highlighting the need for ongoing monitoring and proactive measures to ensure safe marine fish consumption in the Aqaba Gulf.

**Supplementary Information:**

The online version contains supplementary material available at 10.1007/s12011-024-04246-w.

## Introduction

Fish plays a significant role in the nutritional intake of humans and is a vital and excellent supply of omega-3 polyunsaturated fatty acids, minerals, amino acids, and vitamins in comparison to other animal meats and is also easily digested by consumers [1]. Nowadays, greater awareness is being gained about the benefits of eating fish for human health. Furthermore, regular consumption of seafood has been shown to reduce the risk of a number of illnesses, including asthma, thrombosis, heart attacks, strokes, premature births, arrhythmias, and lowering triglyceride levels; hence, it should be ingested at least twice a week. [2–4].

The Egyptian Aqaba Gulf is a significant marine ecosystem commercially. The coastal region of this gulf experienced pollution from cities and industries, port and shipping operations, tourism, and land-based activities such as fertilizer and clinker manufacturing, in addition to the desalination of seawater [5]. Various pollutants may be concentrated more in the Aqaba Gulf region as a result of these operations. Among these pollutants, trace elements pose a major risk to marine habitats due to their poisonous properties.

Trace elements are one of the most prevalent pollutants that marine organisms consume and move through trophic categories via food, sediment, water, or environmental factors [6]. In aquatic environments, fish inhabit the upper trophic levels and have the highest trophic position in the food chain [7]; they are largely responsible for the delivery of contaminants to people [8]. Trace elements accumulate in fish at minor levels via bioaccumulation and at higher concentrations via the biomagnification process; however, people may consume elements via food consumption, which may cause adverse health consequences in the short term [9, 10]. Non-essential elements like Ba, Cr, Pb, Al, As, Cd, and Hg have no biological significance in any manner [11], while essential trace elements like B, Cu, Fe, Mn, Zn, and Ni are required at trace levels for varieties of enzymatic and physiological activities [12, 13]. Elements require a great deal of attention because they cannot be biodegradable, endure for a long time in aquatic systems, and then build up via biomagnification at progressively higher levels throughout the food chain [14].

The bioaccumulation of trace elements in aquatic organisms is regulated by both internal and external variables. Environmental factors that reflect external variables are element bioactivity, temperatures, and the alkalinity of ambient aquatic habitats; internal variables include habitat, size, species, ecology, gender, eating habits, and physiological processes [15]. In the previous years, several studies have documented element pollutants in the carb and bivalve [16], shrimp [17] fish [18–22], crayfish [23, 24] mussel [25], aquatic macrophytes [26, 27] sediment [28–30], and seawater [31, 32].

Possible threats to health associated with trace element consumption have been established for several decades. Trace elements have either non-cancer or cancer risks for people [33]. Trace elements pose risks to human health, including liver dysfunction, kidney disease, and skeletal deformity, due to their prolonged and indecomposable presence within the human visceral organ systems [34]. Therefore, it is imperative to investigate the possible health concerns associated with consuming tainted meals. The following were the study’s principal objectives: (1) measure the levels of trace elements in the sediment, water, and muscular tissue of eight Red Sea fish species that were collected from the Aqaba Gulf at the Egyptian Red Sea coast; (2) identify the factors that lead to the bioaccumulation and biosedimentation of elements; (3) assess the possible environmental impacts of trace elements on the region’s aquatic ecosystems; and (4) ascertain the possible health hazards linked to eating Red Sea fish. In addition, the study aimed to provide baseline data for health and environmental policies to reduce the hazard effects of trace elements pollution along the Aqaba Gulf region while preserving the natural environment and human health.

## Materials and Methods

### Field of Investigation

**Fig. 1:**
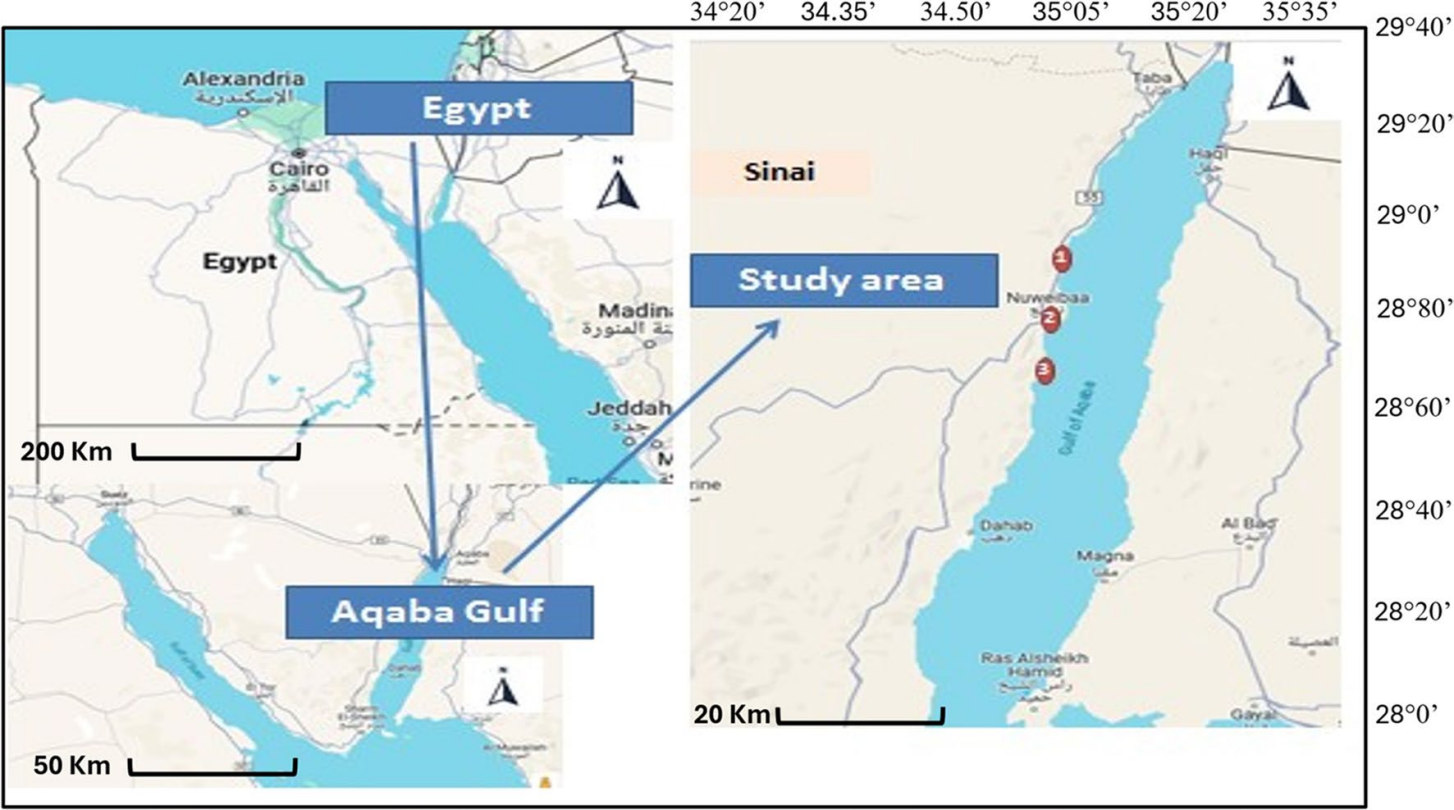
Google map showing the samples’ collection area on Nuweiba City, Aqaba Gulf at the Egyptian Red Sea Coast

The Aqaba Gulf, regarded as one of the most significant aquatic regions in the southern part of the Sinai Peninsula, stretches along the eastern shore of the Red Sea. It is 180 km long, 20 km wide, and 800 m deep. It is situated at 28° N latitudes, 34°23′ E longitudes, and 29°33′ N latitudes, 35°0′ E longitudes [35, 36]. Along the Gulf of Aqaba, there are several towns that are home to a variety of human activities. One such area is Nuweiba City, which is located on the western shore of the Aqaba Gulf. Here, strategic industries and a variety of human activities have an impact on the marine environment in front of the city. Nuweiba City is situated at 28°50′2.7″ N latitude and 34°37′29.6″ E longitude (Fig. 1).

The region of investigation is subject to various sources of pollution, including petrochemical industries, fishing activities, and anthropogenic activities including tourism and ship transformation in Nuweiba port [36, 37]. Marine environment and public health are at risk due to these sources’ elevated levels of trace elements in the sediments, fish, and water. In this regard, studies on trace element concentrations and their impacts on the environment and public health are crucial.

### Sample Collection

Seawater samples were collected at 3 locations (three samples per location) in the Aqaba Gulf using a Ruttner Water Sampler while sediment samples were collected from the same sites using Van Veen Grab Sampler. Eight Red Sea fish were purchased from the local fishermen in Nuweiba City at the collection time when water and sediment samples were collected during October and December 2023. Fish samples (five samples per species, Table 1) were kept in a container with ice and brought to the Faculty of Science laboratory et al.-Azhar University in Egypt. About 10 g of white muscular tissue was removed from the ventral surface anterior to the dorsal fin in the lab. In the lab, the muscular tissues were placed in plastic bags and frozen until analysis.

**Table 1:** Feeding habits, length, and weight of marine fish from Aqaba Gulf at the Egyptian Red Sea Coast

Scientific Name	English name	Local name	Feeding habits	Family	Total length	Stander length	Total weight
*L. ramak*	Snubnose emperor	Bungus	Carnivore	Lethrinidae	23.65 ± 1.83	19.85 ± 1.75	204.31 ± 10.23
*C. hemistiktos*	Groupers	Lucus	Carnivore	Serranidae	29.23 ± 2.08	25.35 ± 1.87	375.11 ± 16.35
*C. suevica*	Suez fusilier	Bagha	Zooplanktivorous	Caesionidae	25.61 ± 2.01	21.35 ± 1.84	197.28 ± 9.52
*C. lunulatus*	Broomtail wrasse	Malaas	Carnivore	Labridae	22.75 ± 2.15	18.95 ± 1.90	240.12 ± 11.36
*P. affinis*	Arabian pandora	Morgan	Carnivore	Sparidae	22.51 ± 1.87	17.83 ± 1.98	215.03 ± 14.21
*T. japonicus*	Horse mackerel	Mackerel	Carnivore	Carangidae	30.14 ± 2.17	27.65 ± 1.70	255.41 ± 15.36
*P. forsskali*	Goatfish	Parpony	Carnivore	Mullidae	24.85 ± 1.08	20.55 ± 1.78	155.96 ± 6.21
*S. luridus*	Marbled Spinefoot	Sigan	Herbivore	Siganidae	20.65 ± 1.07	17.52 ± 0.89	151.45 ± 4.35

No. of samples, *n* = 5 for all species

### Trace Element Measurements

Cd, Al, As, Zn, B, Hg, Ba, Cr, Mn, Fe, Pb, Cu, and Ni levels were measured in water, sediment, and in the muscular tissues of Red Sea fish. Fresh samples weighing about 0.5 g were put in 50-mL digestion tubes containing ultrapure HNO_3_ (65%, 5 mL) and H_2_O_2_ (30%, 1 mL). On the hot plate, the mixture was heated until it was well-digested. Following a period of cooling at ambient temperature, the digested specimens were transferred to volumetric flasks and combined with 1% HNO_3_ to make an ending volume of 25 mL [38]. After filtering the seawater sample using a 0.45-µm filter, 50 mL of samples was acidified with 65% HNO_3_ and 37% HCl, purchased from Merck, Germany, according to APHA [39]. However, the collected sediment was dried overnight at 105 °C, then finely ground, homogenized, and sieved using a 63-µm mesh to select particles smaller than 63 µm. Microwave digestion was used to digest the prepared sample (1.0 g), which was put in a covered Teflon container (Anton-Paar microwave digestion system, Multiwave PRO, with Rotor 8NXF100) with acid mixtures of HNO_3_ (9 mL) and HCl (3 mL) according to EPA method 3051A [40]. Following a period of cooling at ambient temperature, the digested sediment was put in volumetric flasks and combined with deionized water to make an ending volume of 50 mL.

The final solutions of muscles, water, and sediment were determined to have trace elements contents using the ICP-OES technique (inductively coupled plasma optical emission spectrometry, Agilent 5100 series, Australia) and SVDV (synchronous vertical dual view) with continuous flow hydrogen generation and advanced cold vapor systems. The NIST (National Institute of Standards and Technology) provided standard reference material, an external reference, and a quality control sample to verify the accuracy and precision of the trace elements readings. The recoveries of standard reference metals were 90–110%. Trace element concentrations in the fish muscles (on a wet weight basis (ww-b)) and sediment (on a dry weight basis (dw-b)) were measured in µg/g, whereas those in the water were measured in mg/L. The limits of detection (LOD) and limits of quantification (LOQ) of trace elements are shown in Table 1S.

### Bio-concentration Factor Calculation

According to Adolfsson-Erici et al. [41], the bioaccumulation factor (BAF) can be computed as represented in Eq. (1):

1 $$\text{BAF}=\text{M}-\text{fish}/\text{M}-\text{water}$$

where M-fish represents the trace element level in fish muscles (mg/kg), and M-water represents the trace element level in water (mg/L). However, the Eq. (2) [42] was used to compute the biosedimentation factor (BSF):

2 $$\text{BSF}=\text{M}-\text{fish}/\text{M}-\text{sediment}$$

where M-sediment represents the level of trace elements in the sediments (µg/g), and M-fish represents the level of trace elements in the fish muscles (µg/g).

### Environmental Risk Measurement

Numerous indices are used to measure the degree of trace element contamination in aquatic species [43]. In this study, the degree of trace element pollution was assessed using the contamination degree (CD) and the metal pollution index (MPI).

#### Contamination Degree (CD)

Using element levels in the Red Sea fish, the CD was calculated as the following equation (Eq. (3)):

3 $$\text{CD}={C}_{\text{M}}/{C}_{\text{BL}}$$

where *C*_M_ represents trace element level in fish (μg/g ww-b), and *C*_BL_ represents background concentrations of iron, lead, Cd, copper, Ni, and zinc [44].

**Table:** 

CD values	Interpretation
CD ≤ 1	Minimal limit of contamination
1 < CD ≤ 2	Low contamination degree
2 < CD ≤ 3	Moderate contamination
CD > 3	High degree of contamination

#### *Metal Pollution Index (MPI*)

An integrated method for evaluating trace element contamination is MPI. The MPI was estimated using the Eq. (4) [43]:

4 $$\text{MPI}={\left({M}_{1}\times {M}_{2}\times {M}_{3}\times \cdots \cdots \cdots \cdots \times {M}_{x}\right)}^{1/n}$$

where *M*_1_ is the first element level, *M*_2_ is the second element level, *M*_3_ is the third element level, *n* is the number of examined elements and *M*_x_ is the *x*^th^ element level (µg/g ww-b) in the Red Sea fish.

**Table:** 

MPI values	Interpretation
MPI ≤ 1	Safe
2 < CD ≤ 3	Slightly polluted
3 < CD ≤ 5	Moderately polluted
CD > 10	Heavily polluted

### Health Risk Measurement

The potential health risks caused by trace elements ingested by consumption of the muscular tissue of the fish were evaluated according to a method reported by the US Environmental Protection Agency [45]. By measuring trace element levels in the muscles, the estimated daily intake (EDI), non-cancer, and cancer indices were all calculated.

#### Estimated Daily Intake (EDI)

The exposure dosage resulting from direct human eating of certain elements shown in muscular tissue was determined using the EDI (the daily average intake of a certain element over the lifespan). The subsequent equation (Eq. (5)) was used to compute the EDI [46].

5 $$\text{EDI}\left(\text{mg}/\text{kg}/\text{day}\right)=\left[\left(\text{EP}\times \text{IR}\times \text{M}\times \text{ER}\right)/\left(\text{BW}\times \text{AT}\right)\right]\times {10}^{-3}$$

where the EP relates to the lifespan of the exposure period (70 years); the IR denotes the ingestion rate of fish consumption by adults (41 g/day) and children (27 g/day); *M* denotes the trace element levels (μg/g ww-b) in muscular tissue; ER stands for exposure rate (365 days year^−1^); BW denotes the body weight of adults (70 kg) and children (30 kg); and AT (365 days × 70 years) is the average lifespan [45].

#### Target Hazard Quotient (THQ)

The THQ, a non-cancer evaluation of adverse health effects linked to the intake of certain trace element contaminants in muscular tissue, was developed for evaluating human health risk. The ratio of EDI to RfD (oral reference dosage) was employed for determining THQ as in Eq. (6):

6 $$\text{THQ}=\text{EDI}/\text{RfD}$$

The RfD values (mg/kg/day) were Ni (0.02), Cu (0.04), Ba (0.2), Pb (0.00357), Cr (0.003), As (0.003), Mn (0.14), Fe (0.7), and Zn (0.3), in accordance with guidelines provided by USEPA [45].

#### Hazard Index (HI)

According to Cui et al. [47], the HI is an additional computational equation that represents the effect of non-cancer hazards as the total of the THQ values for the trace elements being studied.

7 $$\text{HI}=\sum \text{THQ}$$

#### *Cancer* Risk (CR)

The following formula (Eq. (8)) was used to get the CR values, which represent the incremental risk of developing cancer based on the cancer slope factor [CSF, 48, 49]: 1.7, 0.5, 0.0085, and 1.5 mg/kg/day for Ni, Cr, Pb, and As, respectively.

8 $$\text{CR}=\text{EDI}\times \text{CSF}$$

### Statistical Calculation

The statistical program SPSS (version 22) was used to perform the statistical analyses. To prove homogeneity of variation and a normal distribution, Levene’s test was employed. To find any statistically significant differences (*p* < 0.05) between the trace element levels in the sediment, water, and fish species, analysis of variance (one-way ANOVA) was utilized to statistically examine the results [50]. Also, the correlations between the trace element levels in Red Sea fish were assessed using Pearson’s correlation coefficient. Tables displaying the statistics are formatted as means ± standard deviation.

## Results and Discussion

A significant aquatic environmental issue for aquatic biota is the accumulation of trace elements in aquatic ecosystems because of their increased stability, bioaccumulation, and biomagnification capabilities. Additionally, when these elements move through the food chain, they eventually have an impact on individuals [51]. In aquatic environments, sediments play a crucial role by absorbing and storing a variety of necessary materials as well as hazardous pollutants [52]. Compared to water, analysis of sediment gives accurate estimations for contaminants, which are absorbed by particulates tending to sink on bottoms [53]. It is crucial to get up-to-date information on the levels of toxic trace elements in a variety of Red Sea species in order to comprehend the possible risk of trace elements in these fish to consumers. The concentration of trace elements, such as Cr, Al, Ba, As, B, Cd, Cu, Fe, Pb, Hg, Zn, Mn, and Ni in seawater, sediment, and fish, was determined using the ICP-OES technique.

### Trace Element Levels in Sediment and Water Samples

Table 2 shows the levels of trace elements in the water and sediment of Aqaba Gulf stations. Thirteen trace element levels were reported in the water, and the levels of cadmium and mercury in the sediment were below the detection limit of the ICP-OES technique. Conversely, in water samples, most elements were undetected except for aluminum, boron, iron, and zinc. However, the trace elements in water samples from Aqaba Gulf stations varied from 0.01 ± 0.003 for Zn to 2.86 ± 0.06 mg/l for B elements. Moreover, the maximum levels of elements in sediment samples were recorded for iron (1327.00 ± 524.39 mg/kg), and the minimum levels exhibited 0.53 ± 0.06 mg/kg, for Pb elements. The examined essential trace elements in the water of the Aqaba Gulf were below the allowed level when compared to the acceptable limit [54]. Due to absorption by suspended matter and sediment particles, which quickly remove the trace elements from the water column, the majority of trace elements had low concentrations [55]. Trace element concentrations in the sediment are useful for evaluating pollution trends, and polluted sediment containing trace elements may serve as another source of contamination for the aquatic environment [56]. In the current study, the trace elements studied in the sediment of the Aqaba Gulf were also lower than in the upper continental crust [57].

**Table 2:** The trace element levels in water and sediment from Aqaba Gulf at the Egyptian Red Sea Coast

	Water samples (mg/l)		Sediment samples (µg/g, dw-b)	
	Mean ± SD	WHO 2011	Mean ± SD	Upper continental crust
Non-essential trace elements				
As	BDL		1.20 ± 0.17	
Al	0.11 ± 0.02		908.00 ± 195.42	
Ba	BDL		20.17 ± 7.91	
Pb	BDL		0.53 ± 0.06	17
Cr	BDL		2.63 ± 0.32	
Essential trace elements				
Cu	BDL	2	1.23 ± 0.21	14.3
Fe	0.32 ± 0.25	1	1327.00 ± 524.39	30,980
B	2.86 ± 0.06		4.73 ± 0.32	
Zn	0.01 ± 0.00	3	5.87 ± 2.89	52
Mn	BDL		36.10 ± 16.96	527
Ni	BDL		2.47 ± 1.68	

*T*-test results indicated a significant level (*p* < 0.05) for the different letters between the different ponds in the water and sediment samples

*BDL* below detection limit

#### The Levels of Trace Elements in Red Sea Fish

Fish pollution with trace elements poses a serious risk to both aquatic life and people. Assessing the level of element contamination in muscular tissues begins with measuring the level of trace elements [58]. The levels of trace elements in the muscular tissues of Red Sea samples collected from the Aqaba Gulf are represented in Table 3. The assessment of trace element levels in various fish species, including *L. ramak*, *S. luridus*, *P. forsskali*, *T. japonicus*, *P. affinis*, *and C. lunulatus*, reveals distinctive concentrations of aluminum, arsenic, boron, barium, chromium, copper, iron, lead, zinc, manganese, and nickel in their muscular tissues. In the comprehensive assessment of trace element concentrations within the muscular tissues of various fish species, noteworthy variations were discerned across multiple elements. This intricate pattern of element concentrations within distinct fish species provides valuable insights into the dynamics of trace element accumulation in marine ecosystems. Such variations are to be expected, as the accumulation of trace elements in muscular tissue is dependent on a number of variables, including the trace element concentration in the water, exposure duration, uptake mechanism, and the surrounding environment (pH, dissolved oxygen, temperature). For intrinsic factors, such as habitat, feeding habits, and age [59], several studies have demonstrated that fish trace element levels are mostly dependent on the type of environment they live in [58, 60, 61]. It is widely known that sediment is the primary mechanism for trace element pollution absorption and that it is essential to trace elements [62].

**Table 3:** The trace element levels (µg/g ww-b, Mean ± SD) in marine fish from Aqaba Gulf at the Egyptian Red Sea Coast

	*L. ramak*	*C. hemistiktos*	*C. suevica*	*C. lunulatus*	*P. affinis*	*T. japonicus*	*P. forsskali*	*S. luridus*	*P* value	Permissible limit
Non-essential trace elements										
As	**3.58 ± 0.60**	**1.75 ± 0.30**	**3.95 ± 0.53**	0.48 ± 0.83	**5.00 ± 0.83**	**3.56 ± 0.62**	**5.10 ± 0.79**	**1.35 ± 0.32**	0.006	1 (64)
Al	1.93 ± 0.37	2.95 ± 0.84	4.36 ± 0.93	3.71 ± 0.42	2.00 ± 0.24	2.21 ± 0.33	3.49 ± 0.67	3.87 ± 0.67	0.030	
Ba	0.95 ± 0.65	1.00 ± 0.13	1.71 ± 0.98	2.45 ± 0.80	1.15 ± 0.35	1.02 ± 0.13	1.50 ± 0.14	2.06 ± 0.42	0.034	
Pb	**2.55 ± 0.78**	**3.99 ± 0.80**	**4.53 ± 0.90**	**2.12 ± 1.01**	**3.10 ± 1.01**	**5.86 ± 0.92**	**6.83 ± 0.93**	**3.24 ± 0.78**	0.002	2 (67)
Cr	**2.86 ± 0.96**	**2.26 ± 0.42**	5.25 ± 0.67	1.97 ± 0.46	**2.25 ± 0.35**	**2.29 ± 0.29**	**3.26 ± 0.45**	**2.56 ± 0.42**	0.042	2 (64)
Essential trace elements										
Cu	2.23 ± 0.32	5.76 ± 0.34	6.32 ± 0.87	10.29 ± 1.66	7.80 ± 1.12	4.93 ± 0.50	2.11 ± 0.51	6.84 ± 0.85	0.008	30 (64)
Fe	32.65 ± 2.53	40.66 ± 1.85	81.35 ± 7.62	54.12 ± 2.37	38.00 ± 1.61	31.80 ± 5.94	42.99 ± 1.91	38.27 ± 1.44	0.038	100 (65)
B	5.25 ± 0.58	5.75 ± 0.19	5.18 ± 0.83	6.71 ± 0.92	6.00 ± 0.41	5.88 ± 0.53	6.10 ± 0.17	10.51 ± 0.35	0.005	
Zn	8.14 ± 1.50	7.02 ± 1.61	14.32 ± 1.65	14.36 ± 2.41	7.25 ± 1.06	15.58 ± 1.53	19.75 ± 1.21	11.51 ± 1.00	0.041	40 (65)
Mn	0.72 ± 0.02	0.76 ± 0.07	1.05 ± 0.40	1.31 ± 0.53	0.50 ± 0.03	0.51 ± 0.04	1.25 ± 0.32	0.75 ± 0.03	0.043	30 (65)
Ni	1.20 ± 0.31	1.63 ± 0.40	1.23 ± 0.30	1.51 ± 0.25	1.25 ± 0.35	1.27 ± 0.32	1.76 ± 0.40	1.26 ± 0.39	0.035	2 (66)

Values exceeding the permissible limit are denoted in bold

Arsenic exhibited its peak level at 5.10 ± 0.79 µg/g in *P. forsskali*, juxtaposed with its lowest level of 0.48 ± 0.83 µg/g in *C. lunulatus*. Al displayed its highest level at 4.36 ± 0.93 µg/g in *C. suevica*, while its lowest level was recorded at 1.93 ± 0.37 µg/g in *L. ramak*. Ba reached its zenith in *C. lunulatus* at 2.45 ± 0.80 µg/g, while *L. ramak* manifested the lowest level at 0.95 ± 0.65 µg/g. Pb levels peaked in *P. forsskali* at 6.83 ± 0.93 µg/g and reached their nadir in *C. lunulatus* at 2.12 ± 1.01 µg/g. Cr demonstrated a peak level of 5.25 ± 0.67 µg/g in *C. suevica*, contrasting with its nadir of 1.97 ± 0.46 µg/g in *C. lunulatus*. Cu levels were most elevated in *C. lunulatus* at 10.29 ± 1.66 µg/g and least in *P. forsskali* at 2.11 ± 0.51 µg/g. Fe levels displayed considerable disparity, with the highest observed in *C. suevica* at 81.35 ± 7.62 µg/g and the lowest in *T. japonicus* at 31.80 ± 5.94 µg/g. Moving to boron, *S. luridus* showcased the highest level at 10.51 ± 0.35 µg/g, whereas *C. suevica* featured the lowest at 5.18 ± 0.83 µg/g. Zn levels revealed a wide range, with *P. forsskali* featuring the highest at 19.75 ± 1.21 µg/g and *C. hemistiktos* registering the lowest at 7.02 ± 1.61 µg/g. Manganese (Mn) levels showed a peak of 1.31 ± 0.53 µg/g in *C. lunulatus*, contrasting with a trough of 0.50 ± 0.03 µg/g in *P. affinis*. Finally, nickel (Ni) levels reached their zenith in *P. forsskali* and *C. hemistiktos* (1.76 ± 0.40 µg/g), while *L. ramak* exhibited the lowest at 1.20 ± 0.31 µg/g. In the present study, Zn and Fe were the highest trace elements in the muscles of Red Sea fish, which was in agreement with the result of Younis et al*.* [63].

The essential elements, Al and Ba in the muscular tissues of studied fish were lower than the global acceptable limits provided by different agencies [64–67]. However, the levels of As were higher than the maximum permissible level in all Red Sea fish, except in *C. lunulatus*. Moreover, the levels of Pb in all studied fish were above the uppermost allowable limit. Furthermore, the levels of Cr were higher than the maximum allowable level in all fish, except in *C. lunulatus* and *C. suevica*.

Fish that live close to sediment consume humic materials, and benthic invertebrates collect and supply fish trace elements from the sediment [68]. Consequently, compared to demersal fish, benthic and benthopelagic organisms often show higher levels of trace elements [61]. Additionally, a previous investigation concluded that upper-trophic-level piscivorous organisms appear to collect more trace elements than omnivorous and herbivorous organisms in terms of trace element levels [62], which supports the present study. This result suggests that the trace element levels in aquatic organisms are not only affected by the surrounding environment but also bioaccumulated via their food [68, 69]. A comprehensive overview of trace element concentrations in various fish species, encompassing *L. ramak*, *S. luridus*, *P. forsskali*, *T. japonicus*, *P. affinis*, *C. lunulatus*, *C. suevica*, and *C. hemistiktos*, reveals distinctive patterns in their muscular tissues. Iron consistently dominates with the highest concentration, followed by varying levels of other trace elements. The lowest concentrations are notably found in manganese, nickel, and specific elements depending on the fish species, suggesting potential similarities or differences in element uptake and accumulation mechanisms.

A comparison of the trace element levels (µg/g ww-b) in marine fish species of the present study with the previous studies is represented in Table 4. The level of Al in the current study was lower than those recorded by Mziray and Kimirei [70]. The As level in the current study was within that recorded by Mziray and Kimirei [70] while it was higher than that reported by Al-Amri et al. [71]. The Cr level in the current study was higher than that recorded by [70–72], while it was lower than that reported by Younis et al. [21]. The Cu level in the current study was higher than that recorded by [71–73]. The Fe level in the current study was within that recorded by Mziray and Kimirei [70] and higher than that reported by [71, 72]. The Pb level in the current study was higher than that reported by [21, 58, 70, 72]. The Zn level in the current study was lower than that determined by Mziray and Kimirei [70] and higher than that reported by [21, 58, 71–73]. The Mn level in the current study was lower than that recorded by Mziray and Kimirei [70] and higher than that reported by [21, 72]. The Ni level in the current study was lower than that recorded by Younis et al. [21], while it was higher than that reported by [58, 70–73].

**Table 4:** Comparison between levels of trace element (µg/g ww-b, range) in marine fish species of the present study with the previous studies

		Al	As	Cr	Cu	Fe	Pb	Zn	Mn	Ni
Present study	8 marine species	1.93–4.36	0.48–5.10	1.97–5.25	2.11–10.29	31.80–81.35	2.12–6.83	7.02–19.75	0.50–1.31	1.20–1.76
[73]	*Lethrinus species*	–	–	–	0.07–0.77	–	0.72–4.26	0.01–0.66	–	0.55–1.96
[70]	*Siganus sutor- Lethrinus harak*	19.44–87	3.43–18.52	0.10–0.29	1.65–4.71	34.02–103.29	0.05–0.14	67.8–214.6	5.55–10.18	0.12–0.15
[21]*	5 marine species	–	–	7.63–23.6	1.60–3.94	17.0–39.29	0.15–0.17	–	0.65–1.13	3.60–19.19
[72]*	11 marine species	–	–	0.21–2.08	0.10–0.42	0.52–4.27	0.31–1.73	0.52–7.29	0.10–0.69	0.21–1.04
[58]*	4 marine species	–	–	–	0.61–3.10	–	0.74–1.49	5.78–9.56	–	0.004–0.17
[71]*	14 marine species	–	0.002–0.02	0.01–0.02	0.09–1.31	6.84–20.81	0.00–2.75	0.94–1.34	–	0.00–1.29

^*^Fish dry weight (dw-b) was converted to wet weight (ww-b) using a conversion coefficient of 4.8 [74]

### Trace Element Correlation Coefficient

**Fig. 2:**
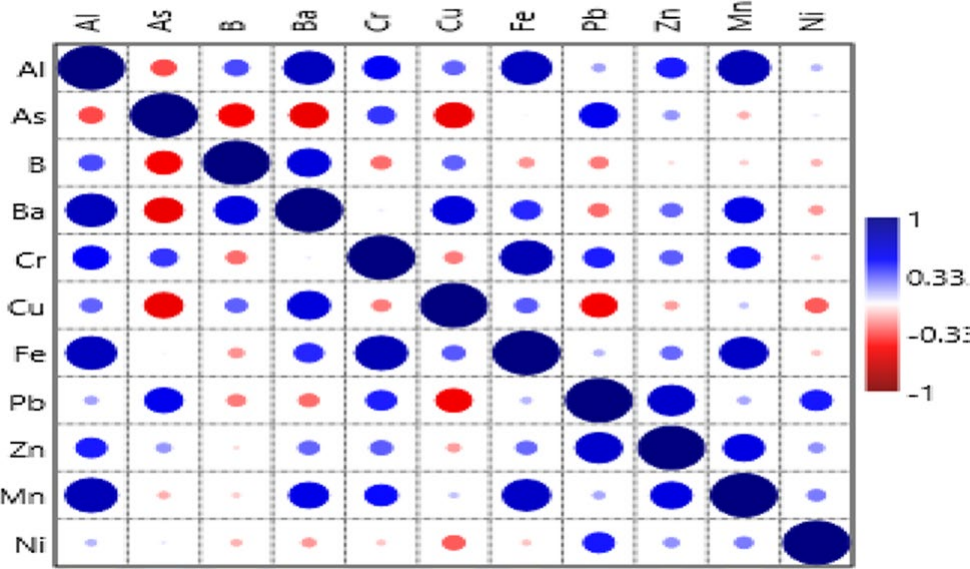
Heatmap showed the correlation coefficient between trace elements in marine fish from Aqaba Gulf at the Egyptian Red Sea Coast

Figure 2 displays the correlation coefficient between trace elements in the muscular tissue of Red Sea fish, as established by the heatmap data. In this study, significant positive correlations were reported for Al with Ba, Cr, Fe, and Mn; As-Pb; Ba-B; Ba-Mn; Mn-Fe, Zn-Pb, and Zn-Mn. According to Yakamercan et al. [75], the positive correlation indicates that the behavior is the same and has a common origin. On the other hand, As was shown to have significant negative associations with B, Ba, and Cu, as well as Cu-Pb. According to Jiang et al. [62], a negative correlation suggests the presence of various sources, mostly dyes and chemicals. Additionally, a hierarchical clustering method according to Euclidean distance was applied to construct relationships for interdependence between the eleven studied trace elements (Fig. 1S). The findings indicated that the ten trace elements were arranged into two major groups (Cu and Zn in cluster 1 and Al-As-B-Ba-Cr-Pb-Mn-Ni in cluster 2), with Fe standing out as belonging to a different group. These findings also indicated that most of the elements originated from the same origins, except for Fe. According to Chai et al. [76], contamination of the marine environment and the transition mechanism of trace elements on different substrates were also significant contributing factors to the origins of elements.

### BAF and BSF Values

**Fig. 3:**
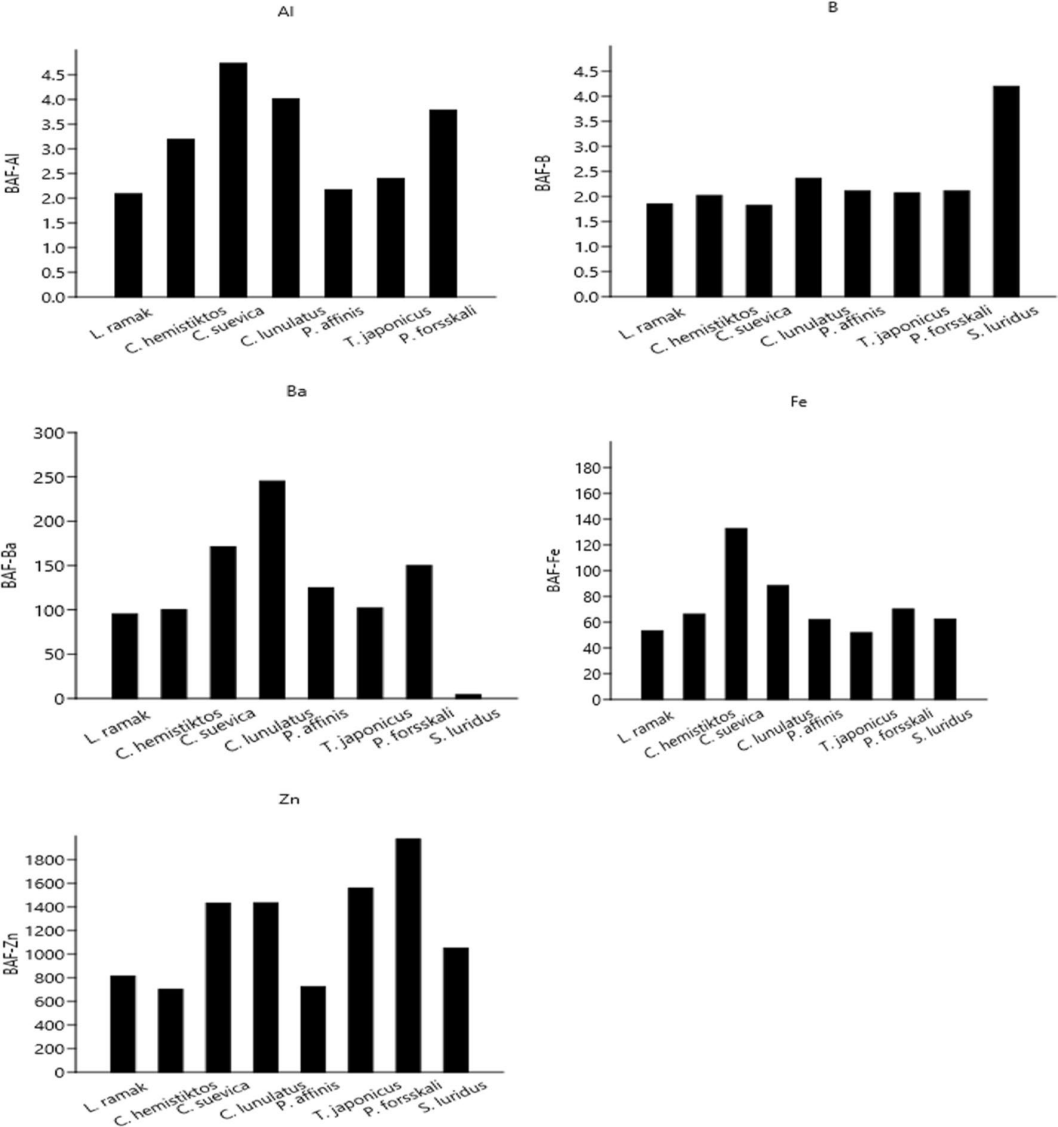
BAF values of trace elements in marine fish from Aqaba Gulf at the Egyptian Red Sea Coast

Bioconcentrations of trace elements associate the number recovered from the portion of collected concentration in a specific organ of different species and the habitat quickly and effectively [77]. The BAF values of trace elements in the Red Sea species are represented in Fig. 3. All studied Red Sea species represented the BAF values in this sequencing order: B < Al < Fe < Ba < Zn.

All species possessed bioaccumulative capability with ascending as BAF-B < BAF-Al < BAF-Fe < BAF-Ba < BAF-Zn were reported in the muscles with the same way of ordination. Among all the species, the BAF value was the highest in *P. forsskali* (1975.3 for BAF-Zn) and the lowest in *C. suevica* (1.82 for BAF-B). In fact, the bioaccumulation of an aquatic species relies on its characteristics, metabolism of inspected tissue, invasion pathways, and habitat condition [78]. Based on the value ranges, BAF can be classified as follows: BAF < 1000: no accumulation probability; 1000 < BAF < 5000: bioaccumulative organism; BAF > 5000: extremely accumulative organism [79]. The current study revealed that BAF-Al, BAF-B, BAF-Ba, and BAF-Fe values in all studied fish were lower than 1000. However, BAF-Zn values in *L. ramak*, *C. hemistiktos*, *P. affinis*, and *S. luridus* were lower than 1000, whereas they were higher than 1000 in *C. suevica*, *C. lunulatus*, *T. japonicas*, and *P. forsskali*, which means these species are bioaccumulative organisms of Zn from water.

**Fig. 4:**
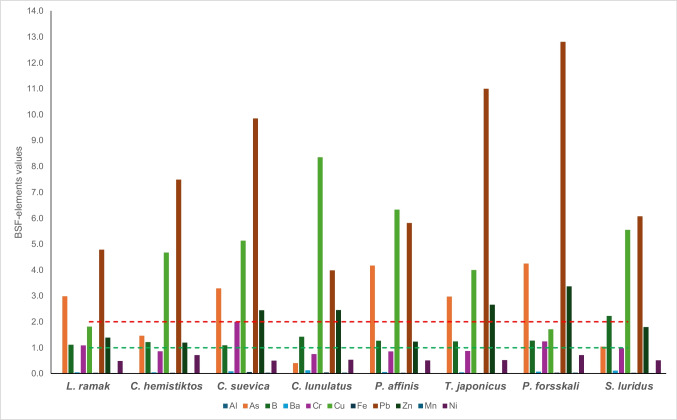
BSF-element values in marine fish from Aqaba Gulf at the Egyptian Red Sea Coast

However, the BSF values based on the studied elements (Al, As, B, Ba, Cr, Cu, Fe, Pb, Zn, Mn, and Ni) in the Red Sea species were recorded in Fig. 4. All the studied Red Sea species exhibited BSF values that were < 1 recorded for Al, Mn, Fe, Ba, and Ni, while they were greater than 2 recorded for Cu and Pb. Fish can be classified according to the values of BSF > 2, 1 < BSF < 2, and BSF < 1 as macro-concentrators, micro-concentrators, and de-concentrators, respectively [80]. In the current study, the BSF-Al, BSF-Ba, BSF-Mn, and BSF-Ni were < 1 in all fish species, whereas the BSF-Pb was > 2 in all Red Sea fish. However, the BSF-Zn values were > 2 in all species except *L. ramak* (1.39), *C. hemistiktos* (1.20), *P. affinis* (1.24), and *S. luridus* (1.79). Moreover, BSF-As values were > 2 in all fish except *C. hemistiktos* (1.45), *C. lunulatus* (0.40), and *S. luridus* (1.04). Additionally, the BSF-B values were 1 < BSAF < 2 in all species except *S. luridus* (2.22). Furthermore, the BSF-Cr values were lower than 1 in all species except *L. ramak* (1.09), *C. suevica* (2.00), and *P. forsskali* (1.24). On the other hand, the BSF-Cu values were > 2 in all fish except *L. ramak* (1.81) and *P. forsskali* (1.71).

### PCA Analysis

In PCA, the components were taken into account whose eigenvalues were greater than 0.5 (Table 5). PCA explained 99.73% of the data variation, and a total of 8 significant PCs were extracted with an eigenvalue > 1. PC1 explained 88.27% of the total variances and exhibited an eigenvalue of 268.379. PC1 was dominated by Fe, with loadings of 0.99. The employed PCA revealed that the source of origin of the element was anthropogenic. B, As, Al, Cu, Pb, Ni, and Fe were the dominant compounds in PCA analysis due to their high loading scores in their respective components. PC2 explained around 7.36% of the total variance, and Zn contained the highest loading scores (0.89). Besides, PC3 explained 2.84% of the total variance and was dominated by Cu (0.67), whereas PC4 explained 0.82% of the total variance with a maximum loading of B (0.68). PC5 explained 0.45% of the total variance with a maximum loading of As (0.68) and B (0.50). The loadings of As and Pb are very close in PC5, which represents a similar source of these elements. However, PC6 explained around 7.82% and 6% of the total variance with the maximum loading of Zn (0.55) and Fe (0.67), respectively. Besides, PC7 explained 0.23% of the total variance with a maximum loading of Pb (0.82), and PC8 explained 0.04% of the total variance with a moderately favorable loading of Ni (0.52).

**Table 5:** Component matrix of eight-factor model with moderate loadings in fish

	PC 1	PC 2	PC 3	PC 4	PC 5	PC 6	PC 7
Non-essential trace elements							
As	− 0.002	0.149	− 0.412	− 0.326	0.680	− 0.419	0.232
Al	0.043	0.030	0.123	0.267	0.022	0.111	0.504
Ba	0.015	− 0.003	0.158	0.098	0.026	− 0.199	0.211
Pb	0.016	0.288	− 0.172	0.019	0.379	0.821	− 0.034
Cr	0.051	0.047	− 0.171	0.174	0.126	− 0.098	− 0.464
EDI-essential trace elements							
Cu	0.054	− 0.298	0.667	− 0.551	0.311	0.127	− 0.055
Fe	0.992	− 0.074	− 0.059	0.033	− 0.007	− 0.005	0.001
B	− 0.022	− 0.047	0.384	0.681	0.507	− 0.139	− 0.077
Zn	0.090	0.891	0.368	− 0.116	− 0.109	− 0.161	− 0.062
Mn	0.015	0.025	0.024	0.025	− 0.098	− 0.070	0.384
Ni	− 0.002	0.016	− 0.010	0.013	− 0.031	0.151	0.521
Eigenvalue	268.379	22.372	8.632	2.493	1.352	0.708	0.113
% variance	88.268	7.358	2.839	0.820	0.445	0.233	0.037
Cumulative variance %	88.27	95.63	98.47	99.29	99.73	99.96	100

Extraction method: Principal Component Analysis (PCA)

Moderate loading value (> 0.5)

### Environmental Risk Assessment

**Fig. 5:**
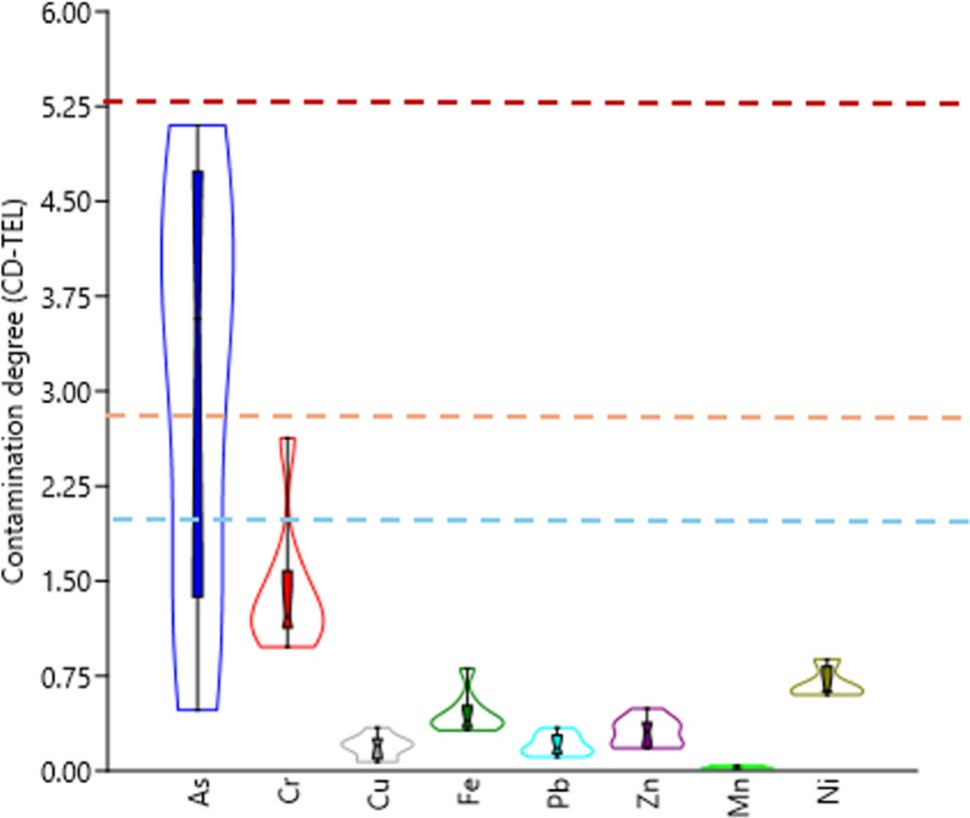
The CD values of trace elements in marine fish from Aqaba Gulf at the Egyptian Red Sea Coast

**Fig. 6:**
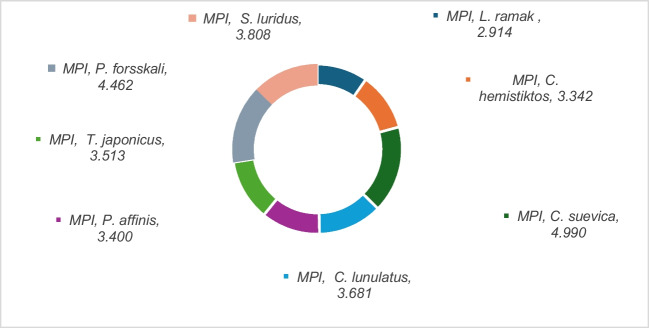
The MPI values of trace elements in marine fish from Aqaba Gulf at the Egyptian Red Sea Coast

The degree of contamination was evaluated by the contamination factor and the element pollution index [81]. The contamination factor (CD) and element pollution index (MPI) were represented in Figs. 5 and 6, respectively.

#### Contamination Degree (CD)

The CD values were calculated based on the levels of As, Cr, Cu, Fe, Pb, Zn, Mn, and Ni. However, the contamination factors for Cu, Fe, Pb, Zn, Mn, and Ni were lower than 1, which indicates a minimal limit of contamination in the Red Sea fish. Moreover, Cf-As in *C. lunulatus*, *S. luridus*, and *C. hemistiktos* were ≥ 1, while the remaining species recorded a high degree of contamination (CD-As > 3), which was ordered in the following sequence: *T. japonicus* (3.56) < *L. ramak* (3.58) < *C. suevica* (3.95) < *P. affinis* (5.00) < *P. forsskali* (5.10). On the other hand, the contamination degrees of Cr were denoted as a low contamination degree (1 < CD-Cr ≤ 2) in all studied fish samples except *C. suevica* (2.63), which was denoted as a moderate contamination degree. The CD values indicate the contamination level. The calculated CD values for Cu, Fe, Pb, Zn, Mn, and Ni were < 1, which indicates a minimal limit of contamination in the Red Sea fish (Fig. 5).

#### Element Pollution Index (MPI)

MPI is generally used to define the polluted degree of trace elements in the tissues of fish. It is considered that the higher value of the estimated MPI describes the higher degree of contamination in fish [82]. The MPI based on elements (Al, As, B, Ba, Cd, Cr, Cu, Fe, Pb, Zn, Hg, Mn, and Ni) in the muscles of nine Red Sea species is presented in Fig. 6. MPI varied from 2.63 in *L. ramak* to 4.63 in *C. suevica* and was severely contaminated. However, it was higher than 1 for CD-As and CD-Cr in the studied fish except in *C. lunulatus*. However, MPI compared total element levels between samples [83]. Hence, the more contaminated samples have higher MPI levels [42, 82]. In the present study, the contamination degree based on MPI values of trace elements in studied fishes can be classified as follows: *L. ramak* (2.63) < *C. hemistiktos* (3.10), < *P. affinis* (3.16), < (3.17) < *T. japonicus* (3.25), *C. lunulatus* (3.43) < *S. luridus* (3.50), < *P. forsskali* (3.92) < *C. suevica* (4.63). Low levels of MPI in the muscle might be related to low metabolic activity and low element-binding proteins in this muscle [84, 85]. The highest MPI value was obtained for *C. suevica* and the lowest for *L. ramak*. MPI was considered using trace element concentrations in the fish species and was used to compare the total element contents of the muscle of the examined fish.

### Human Health Risk Assessment

Fish are one of the main sources of protein for the human diet worldwide, but they are also known as being the largest bioaccumulators of trace elements due to their position at the top of the food web and food chain in both freshwater [86] and marine ecosystems [87]. The high content of elements, including both transitional elements and elementloids, can have potential adverse effects on human health and the environment. However, it is important to note that to select a bioindicator species to assess human health effects, it should meet certain criteria, such as being commonly consumed in the area, widely distributed geographically, having the potential to accumulate high element concentrations, and having adequate tissue mass for residue analysis [88].

#### Estimated Daily Intake (EDI)

Trace elements of poisonous quality could incline people to health hazards through the consumption of defiled aquatic food; therefore, it is crucial for assessment [16]. Based on the oral reference dose RFD, the values of EDI were calculated to measure both the non-cancer and cancer risks of element consumption through seafood [89]. Besides, the EDI value denotes the exposure of trace elements, which is performed to evade any detrimental impact on human health [90]. Consumers’ daily exposure to trace elements through eating foods high in trace elements was employed to avoid any detrimental effects on humans during their lifespan [91]. The values of EDI were explored from selected 11 elements: Al, As, B, Ba, Cd, Cr, Cu, Fe, Pb, Zn, Hg, Mn, and Ni on the Red Sea fish consumption by children and adult consumers, as documented in Table 6. In addition, EDI was calculated, emphasizing the oral reference dose (RfD) for a specific chemical that elaborates the everyday exposition to noxious components and avoids any deleterious outcome on human health over lifetime exposure. The recorded EDI values were higher for child consumers compared to adult consumers, indicating that children are possibly more vulnerable to the health risks related to consuming trace elements in Red Sea fish. Other studies [75, 92, 93] also observed a similar pattern.

**Table 6:** EDI values in marine fish from Aqaba Gulf at the Egyptian Red Sea Coast

		*L. ramak*	*C. emistiktos*	*C. suevica*	*C. lunulatus*	*P. affinis*	*T. japonicus*	*P. forsskali*	*S. luridus*	[94]
EDI-non-essential trace elements										
As	Children	3.2E − 03	1.6E − 03	3.6E − 03	4.3E − 04	4.5E − 03	3.2E − 03	4.6E − 03	1.1E − 03	2E + 00
	Adults	2.1E − 03	1.0E − 03	2.3E − 03	2.8E − 04	2.9E − 03	2.1E − 03	8.8E − 03	7.3E − 04	
Al	Children	1.7E − 03	2.7E − 03	3.9E − 03	3.3E − 03	1.8E − 03	2.0E − 03	3.1E − 03	3.5E − 03	1E + 00
	Adults	1.1E − 03	1.7E − 03	2.6E − 03	2.2E − 03	1.2E − 03	1.3E − 03	2.0E − 03	2.3E − 03	
Ba	Children	8.6E − 04	9.0E − 04	1.5E − 03	2.2E − 03	1.1E − 03	9.2E − 04	1.4E − 03	2.0E − 03	2.0E − 01
	Adults	5.6E − 04	5.9E − 04	1.0E − 03	1.4E − 03	7.3E − 04	6.0E − 04	8.8E − 04	1.3E − 03	
Pb	Children	2.3E − 03	3.6E − 03	4.7E − 03	1.9E − 03	2.8E − 03	5.3E − 03	6.1E − 03	2.9E − 03	3E − 02
	Adults	1.5E − 03	2.3E − 03	3.1E − 03	1.2E − 03	1.8E − 03	3.4E − 03	4.0E − 03	1.9E − 03	
Cr	Children	2.6E − 03	2.0E − 03	4.7E − 03	1.8E − 03	2.0E − 03	2.1E − 03	2.9E − 03	2.3E − 03	2E − 01
	Adults	1.7E − 03	1.3E − 03	3.1E − 03	1.2E − 03	1.3E − 03	1.3E − 03	1.9E − 03	1.5E − 03	
EDI-essential trace elements										
Cu	Children	2.0E − 03	5.2E − 03	5.7E − 03	9.3E − 03	7.0E − 03	4.4E − 03	1.9E − 03	6.2E − 03	5E + 01
	Adults	4.2E − 04	1.5E − 03	1.6E − 03	2.8E − 03	2.1E − 03	1.2E − 03	2.9E − 04	1.8E − 03	
Fe	Children	2.9E − 02	3.7E − 02	7.3E − 02	4.9E − 02	3.4E − 02	2.9E − 02	3.9E − 02	3.4E − 02	5E + 01
	Adults	1.9E − 02	2.4E − 02	4.8E − 02	3.2E − 02	2.2E − 02	1.9E − 02	2.5E − 02	2.2E − 02	
B	Children	4.7E − 03	5.2E − 03	4.7E − 03	6.0E − 03	5.4E − 03	5.3E − 03	5.4E − 03	9.5E − 03	
	Adults	3.1E − 03	3.4E − 03	3.0E − 03	3.9E − 03	3.5E − 03	3.4E − 03	3.5E − 03	6.2E − 03	
Zn	Children	7.3E − 03	6.3E − 03	1.3E − 02	1.3E − 02	6.5E − 03	1.4E − 02	1.8E − 02	9.5E − 03	7E + 01
	Adults	4.8E − 03	4.1E − 03	8.4E − 03	8.4E − 03	4.2E − 03	9.1E − 03	1.2E − 02	6.2E − 03	
Mn	Children	6.5E − 04	6.8E − 04	1.1E − 03	1.2E − 03	4.5E − 04	4.6E − 04	1.1E − 03	6.7E − 04	14E + 01
	Adults	4.2E − 04	4.4E − 04	7.3E − 04	7.7E − 04	2.9E − 04	3.0E − 04	7.3E − 04	4.4E − 04	
Ni	Children	1.1E − 03	1.6E − 03	1.1E − 03	1.2E − 03	1.1E − 03	1.1E − 03	1.6E − 03	1.1E − 03	4E − 02
	Adults	7.0E − 04	1.0E − 03	7.2E − 04	7.7E − 04	7.3E − 04	7.4E − 04	1.0E − 03	7.4E − 04	

However, the EDI values for trace elements show a range of intake levels for children and adults. The EDI values (mg kg^−1^ day^−1^) of non-essential Al, As, Ba, Cd, Pb, Cr, and Hg and essential trace elements Cu, Fe, B, Zn, Mn, and Ni on Red Sea fish consumption by children and adult consumers were lower than the PTDI (permissible tolerable daily intake [94]). Therefore, the lower EDI than the permissible guidelines revealed that there is a possibility of a non-health impact associated with trace elements on consumers; mostly children would be more susceptible.

#### Target Hazard Quotient (THQ)

The allowable threshold level of THQ is one [45]. THQ for 11 elements: Al, As, B, Ba, Cd, Cr, Cu, Fe, Pb, Zn, Hg, Mn, and Ni in the muscles of Red Sea fish is illustrated in Table 7. However, the estimated THQ values were also higher in the child group than in the adult group for all studied elements. The THQ-essential elements, THQ-Al, and THQ-Ni values determined in edible Red Sea fish were under 1 [95], suggesting that eating muscles will not have any adverse health effects for consumers who consumed the studied Red Sea fish. However, the THQ-Cr values in the studied fish were also lower than 1, except in *C. suevica* for both consumers. The THQ-A values for adult consumers were < 1, except for *P. forsskali* (2.95), while they were higher than 1 for children consumers, only in *L. ramak* (1.08) > *C. suevica* (1.18) > *P. affinis* (1.50) > *T. japonicus* (1.07) > *P. forsskali* (1.53). Furthermore, the THQ-Pb values for adult consumers were < 1, except in *P. forsskali* (1.12), while they were higher than 1 for children consumers, only in *C. hemistiktos* (1.01), *C. suevica* (1.32), *T. japonicus* (1.48), and *P. forsskali* (1.72).

**Table 7:** THQ values of trace elements in marine fish from Aqaba Gulf at the Egyptian Red Sea Coast

		*L. ramak*	*C. emistiktos*	*C. suevica*	*C. lunulatus*	*P. affinis*	*T. japonicus*	*P. forsskali*	*S. luridus*
THQ-non-essential trace elements									
As	Children	**1.1E + 00**	5.2E − 01	**1.2E + 00**	1.4E − 01	**1.5E + 00**	**1.1E + 00**	**1.5E + 00**	3.7E − 01
	Adults	7.0E − 01	3.4E − 01	7.7E − 01	9.4E − 02	9.8E − 01	7.0E − 01	**2.9E + 00**	2.4E − 01
Al	Children	1.7E − 03	2.7E − 03	3.9E − 03	3.3E − 03	1.8E − 03	2.0E − 03	3.1E − 03	3.5E − 03
	Adults	1.1E − 03	1.7E − 03	2.6E − 03	2.2E − 03	1.2E − 03	1.3E − 03	2.0E − 03	2.3E − 03
Ba	Children	4.3E − 03	4.5E − 03	7.7E − 03	1.1E − 02	5.6E − 03	4.6E − 03	6.8E − 03	1.0E − 02
	Adults	2.8E − 03	2.9E − 03	5.0E − 03	7.2E − 03	3.7E − 03	3.0E − 03	4.4E − 03	6.6E − 03
Pb	Children	6.4E − 01	**1.0E + 00**	**1.3E + 00**	5.4E − 01	7.8E − 01	**1.5E + 00**	**1.7E + 00**	8.2E − 01
	Adults	4.2E − 01	6.6E − 01	8.6E − 01	3.5E − 01	5.1E − 01	9.6E − 01	**1.1E + 00**	5.3E − 01
Cr	Children	8.6E − 01	6.8E − 01	**1.6E + 00**	5.9E − 01	6.8E − 01	6.9E − 01	9.8E − 01	7.7E − 01
	Adults	5.6E − 01	4.4E − 01	**1.0E + 00**	3.8E − 01	4.4E − 01	4.5E − 01	6.4E − 01	5.0E − 01
THQ-essential trace elements									
Cu	Children	5.0E − 02	1.3E − 01	1.4E − 01	2.3E − 01	1.8E − 01	1.1E − 01	4.7E − 02	1.5E − 01
	Adults	1.0E − 02	3.6E − 02	4.0E − 02	7.0E − 02	5.1E − 02	3.0E − 02	7.3E − 03	4.4E − 02
Fe	Children	4.2E − 02	5.2E − 02	1.0E − 01	7.0E − 02	4.9E − 02	4.1E − 02	5.5E − 02	4.9E − 02
	Adults	2.7E − 02	3.4E − 02	6.8E − 02	4.5E − 02	3.2E − 02	2.7E − 02	3.6E − 02	3.2E − 02
B	Children	2.8E − 02	3.0E − 02	2.7E − 02	3.6E − 02	3.2E − 02	3.1E − 02	3.2E − 02	5.6E − 02
	Adults	1.8E − 02	2.0E − 02	1.8E − 02	2.3E − 02	2.1E − 02	2.0E − 02	2.1E − 02	3.6E − 02
Zn	Children	2.4E − 02	2.1E − 02	4.3E − 02	4.3E − 02	2.2E − 02	4.7E − 02	5.9E − 02	3.2E − 02
	Adults	1.6E − 02	1.4E − 02	2.8E − 02	2.8E − 02	1.4E − 02	3.0E − 02	3.9E − 02	2.1E − 02
Mn	Children	4.6E − 03	4.9E − 03	8.0E − 03	8.4E − 03	3.2E − 03	3.3E − 03	8.0E − 03	4.8E − 03
	Adults	3.0E − 03	3.2E − 03	5.2E − 03	5.5E − 03	2.1E − 03	2.1E − 03	5.2E − 03	3.1E − 03
Ni	Children	5.4E − 02	7.9E − 02	5.5E − 02	5.9E − 02	5.6E − 02	5.7E − 02	7.9E − 02	5.6E − 02
	Adults	3.5E − 02	5.1E − 02	3.6E − 02	3.8E − 02	3.7E − 02	3.7E − 02	5.1E − 02	3.7E − 02

Values exceeding the permissible limit are denoted in bold

#### Hazard Index (HI)

**Fig. 7:**
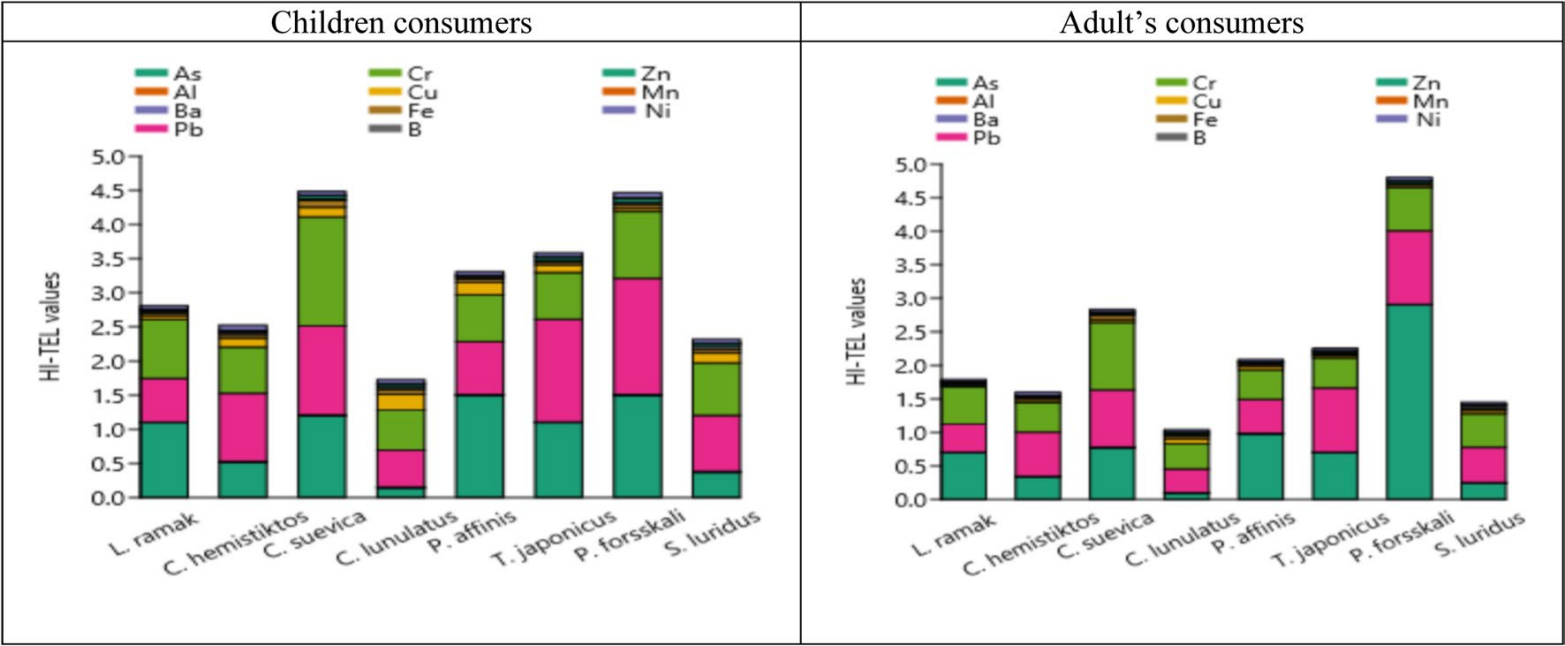
The HI values of 11 trace elements in marine fish from Aqaba Gulf at the Egyptian Red Sea Coast

The hazard index (HI) data for trace elements in nine Red Sea fish species shows a range of values for children and adults (Fig. 7). HI values for both adults and children through consumption of the eleven Red Sea fish species were evaluated based on the THQ values; if the HI value was higher than ten (HI > 10), the exposed consumer would face a significant non-cancer health risk, as recommended by Saha et al. [96]. The arranged hazard index (HI) values for children and adult consumers based on the types of fish are as follows: *C. lunulatus* (1.73 and 1.05, respectively) < *S. luridus* (2.32 and 1.46, respectively) < *C. hemistiktos* (2.53 and 1.60, respectively) < *L. ramak* (2.78 and 1.79, respectively) < *P. affinis* (3.30 and 2.09, respectively) < *T. japonicus* (3.53 and 2.26, respectively) < *C. suevica* (4.48 and 2.86, respectively) < *P. forsskali* (4.52 and 4.87, respectively)*.* The HI values in the muscles of fish were less than ten, suggesting no non-cancer hazard for human consumption occurred.

#### Cancer Risk (CR) Values

**Fig. 8:**
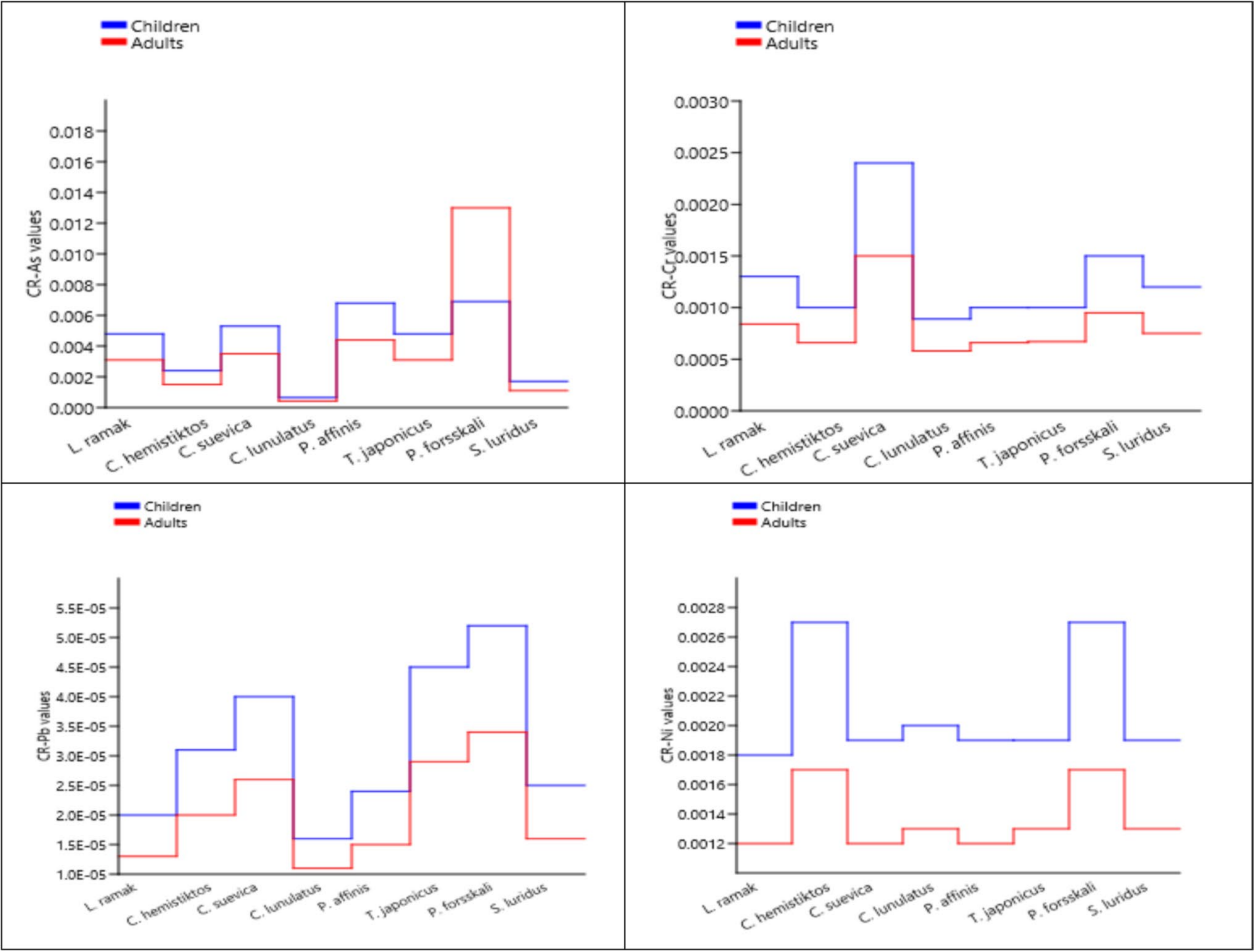
The cancer risk (CR) values in marine fish from Aqaba Gulf at the Egyptian Red Sea Coast

The cancer risk values of trace elements in the muscles of Red Sea fish vary across different species for children and adults (Fig. 8). The maximum values of CR-As for both children and adults were detected in *P. forsskali* (6.5E − 04 and 4.2E − 04, respectively) while the minimum values were determined in *C. lunulatus* (6.9E − 03 and 1.3E − 02, respectively).

CR values lower than 10^−6^ denote the elements’ negligible exposure, whereas 10^−6^ to 10^−4^ means the acceptable range, and higher than 10^−4^ indicates terrible exposure [74]. Estimated CR-As, CR-Ni, CR-Cr, and CR-Pb values in most studied species were higher in children than adults. For both consumers, the CR-Pb values in all studied Red Sea fish were within the limit of 10^−4^ to 10^−6^, though the other cancer risk values (CR-As, CR-Cr, and CR-Ni) showed higher cancer risk than the permissible limit as the increasing sequencing of CR-As > CR-Ni > CR-Cr. The CR values for children and adult consumers were ordered as the sequencing of CR-Pb < CR-Cr < CR-As < CR-Ni in all studied Red Sea fishes except *C. lunulatus*, which showed this sequencing: CR-Pb < CR-As < CR-Cr < CR-Ni.

## Conclusion

Trace element pollution is a global problem that has implications for ecosystem functioning, global food safety, and global security. While extensive studies on trace element levels in marine fish have been carried out in many parts of the world to guide marine fish consumption, little or no studies exist in other parts of the world, including the Gulf of Aqaba Region. The study, therefore, aims to fill an important knowledge gap by providing information on thirteen trace element levels in eight (8) consumed marine fish species from the present Gulf and their possible implications for human health. Thirteen trace elements were analyzed in the sediment, water, and muscle of Red Sea fish from Nuweiba City, Aqaba Gulf, Egypt. The consumption of these polluted fish causes potential health hazards for its consumers. This study concluded that various trace elements accumulated at various concentrations in different fish species. The results exhibited that the bioaccumulation of essential elements in the muscle is well within the safe level as recommended by national and international agencies. However, the results of the non-essential trace elements in the present study indicated that several studied fish species could not be totally safe for human consumption due to their associated cancer and non-cancer potential health risks. An understanding of the adverse effects of trace elements on Red Sea fish and their permissible concentrations in aquatic environments would be extremely essential for fish conservation, fisheries development, and safe human consumption. Overall, monitoring trace element accumulation in Red Sea fish and assessing its health implications are crucial for human well-being.

## Supplementary Information

Below is the link to the electronic supplementary material.

Supplementary file1 (DOCX 25.5 KB)

## Data Availability

The authors declare that data are available from the corresponding author at the readers’ request.
