# Supplementary material for: Elucidating the Environmental and Health Risks of Trace Element Pollution in Red Sea Fish from Nuweiba City, Aqaba Gulf, Egypt
Source: Biol Trace Elem Res. 2024 Jun 28;203(3):1618–36. doi: 10.1007/s12011-024-04246-w (PMC11872992; doi:10.1007/s12011-024-04246-w)
Supplement: Supplementary file 1 — Supplementary file1 (DOCX 25.5 KB) [file 12011_2024_4246_MOESM1_ESM.docx]

Elucidating the Environmental and Health Risks of Trace Element Pollution in Red Sea Fish from Nuweiba City, Aqaba Gulf, Egypt

**Mohamed A. El- Shorbagy^1^, Shimaa M. Abdel-Moniem^2^, Mohamed H. Ghanem^1^, Mohamed A. Embaby****^3^, Mohamed S. Kourany^4^, Ahmed A. El-Kady^3^,** **Mahmoud Mahrous M. Abbas^1*^**

^1^Marine Biology branch, Zoology Department, Faculty of Science, Al-Azhar University, Cairo, Egypt.

^2^Water Pollution Research Department, Environmental and Climate Changes Research Institute, National Research Centre, Cairo, Egypt.

^3^Food Toxicology and Contaminants Department, National Research Centre, Cairo, Egypt.

^4^Food Science and Technology Department, Agriculture Faculty, Fayoum University, Fayoum, Egypt.

*Corresponding author: **E-Mail:** [Mahmoud_Mahrous42@azhar.edu.eg](mailto:Mahmoud_Mahrous42@azhar.edu.eg%20)

**Phone:** 00201064303459 **ORCID:** <https://orcid.org/0000-0002-2061-4101>

| **Metals** | **LOD (mg/L)** | **LOQ (mg/L)** |
| --- | --- | --- |
| **Cadmium** | 0.002 | 0.0076 |
| **Lead** | 0.0077 | 0.026 |
| **Chromium** | 0.007 | 0.025 |
| **Nickel** | 0.0066 | 0.025 |
| **Iron** | 0.0029 | 0.0095 |
| **Manganese** | 0.0029 | 0.0095 |
| **Copper** | 0.025 | 0.084 |
| **Zinc** | 0.0029 | 0.0096 |
| **Mercury** | 0.002 | 0.0065 |
| **Arsenic** | 0.001 | 0.0035 |
| **Boron** | 0.01 | 0.037 |
| **Barium** | 0.01 | 0.035 |
| **Aluminum** | 0.01 | 0.03 |

Table 1S. The limits of detection (LOD) and limits of quantification (LOQ) of trace elements.


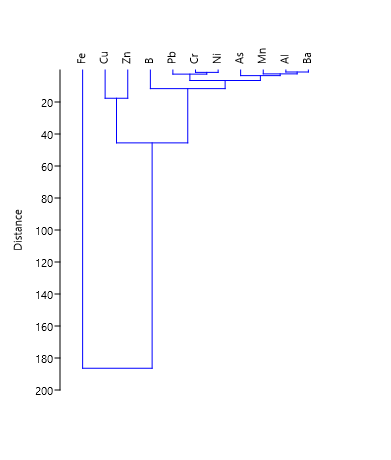


Fig. S1. Hierarchical cluster analysis (Dendrogram) of trace elements in marine fish from Aqaba Gulf at Egyptian Red Sea Coast.
